# Inhibiting leukocyte‐endothelial cell interactions by Chinese medicine Tongxinluo capsule alleviates no‐reflow after arterial recanalization in ischemic stroke

**DOI:** 10.1111/cns.14242

**Published:** 2023-04-30

**Authors:** Shen Liu, Zhaoxu Zhang, Yannan He, Lingbo Kong, Qiushuo Jin, Xiangjia Qi, Dahe Qi, Ying Gao

**Affiliations:** ^1^ Department of Neurology of TCM, Dongzhimen Hospital Beijing University of Chinese Medicine Beijing China; ^2^ Department of Rehabilitation Medicine The First Affiliated Hospital of Shandong First Medical University and Shandong Provincial Qianfoshan Hospital Jinan China; ^3^ Department of Neurology Peking University People's Hospital Beijing China; ^4^ Key Laboratory of Chinese Internal Medicine of Ministry of Education, Dongzhimen Hospital Beijing University of Chinese Medicine Beijing China; ^5^ Department of Neurology The First Affiliated Hospital of Shandong First Medical University and Shandong Provincial Qianfoshan Hospital Jinan China; ^6^ Institute for Brain Disorders, Beijing University of Chinese Medicine Beijing China

**Keywords:** inflammatory factors, ischemic stroke, leukocyte‐endothelial cell interactions, no‐reflow, Tongxinluo capsule

## Abstract

**Aims:**

Despite successful vascular recanalization in stroke, one‐fourth of patients have an unfavorable outcome due to no‐reflow. The pathogenesis of no‐reflow is fully unclear, and therapeutic strategies are lacking. Upon traditional Chinese medicine, Tongxinluo capsule (TXL) is a potential therapeutic agent for no‐reflow. Thus, this study is aimed to investigate the pathogenesis of no‐reflow in stroke, and whether TXL could alleviate no‐reflow as well as its potential mechanisms of action.

**Methods:**

Mice were orally administered with TXL (3.0 g/kg/d) after transient middle cerebral artery occlusion. We examined the following parameters: neurological function, no‐reflow, leukocyte‐endothelial cell interactions, HE staining, leukocyte subtypes, adhesion molecules, and chemokines.

**Results:**

Our results showed stroke caused neurological deficits, neuron death, and no‐reflow. Adherent and aggregated leukocytes obstructed microvessels as well as leukocyte infiltration in ischemic brain. Leukocyte subtypes changed after stroke mainly including neutrophils, lymphocytes, regulatory T cells, suppressor T cells, helper T type 1 (Th1) cells, Th2 cells, B cells, macrophages, natural killer cells, and dendritic cells. Stroke resulted in upregulated expression of adhesion molecules (P‐selectin, E‐selectin, and ICAM‐1) and chemokines (CC‐chemokine ligand (CCL)‐2, CCL‐3, CCL‐4, CCL‐5, and chemokine C‐X‐C ligand 1 (CXCL‐1)). Notably, TXL improved neurological deficits, protected neurons, alleviated no‐reflow and leukocyte‐endothelial cell interactions, regulated multiple leukocyte subtypes, and inhibited the expression of various inflammatory mediators.

**Conclusion:**

Leukocyte‐endothelial cell interactions mediated by multiple inflammatory factors are an important cause of no‐reflow in stroke. Accordingly, TXL could alleviate no‐reflow via suppressing the interactions through modulating various leukocyte subtypes and inhibiting the expression of multiple inflammatory mediators.

## Graphical abstract

Leukocyte‐endothelial cell interactions mediated by multiple adhesion molecules and chemokines are an important cause of no‐reflow after arterial recanalization in ischemic stroke. Chinese medicine Tongxinluo capsule could alleviate no‐reflow via suppressing the interactions through modulating various leukocyte subtypes and inhibiting the expression of multiple inflammatory mediators.

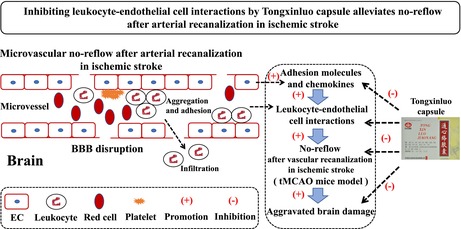

## 1 INTRODUCTION

In recent years, vascular recanalization resulting from intravenous thrombolysis and/or intravascular interventional therapy represents a significant breakthrough in the treatment of acute ischemic stroke. However, 50% of stroke patients with successful arterial recanalization have an unfavorable outcome.^1^ Namely, the recanalization does not equate with successful tissue reperfusion due to the fact that part of the recanalization is futile.^2^ The journal *Stroke* has reported that a quarter of patients with successful arterial recanalization in ischemic stroke has impaired tissue reperfusion, termed as no‐reflow phenomenon.^3^ Of note, the phenomenon has become one of the main bottlenecks of constraining further improvement of clinical efficacy of ischemic stroke. Currently, its underlying mechanisms have not been fully understood, and effective therapeutic strategies are lacking in western medicine.^1^

Based on the collateral disease theory of traditional Chinese medicine (TCM), minute collaterals in TCM and microvessels in western medicine are highly correlated in morphology and function; it is then considered that the main pathological mechanism of no‐reflow is minute collateral‐microvessel obstruction. Thus, we propose that unblocking‐collateral intervention is its therapeutic principle according to the theory. It has been confirmed that Tongxinluo capsule (TXL), a representative Chinese medicine based upon unblocking‐collateral therapy can unblock collaterals and protect microvessels in ischemic cardiovascular and cerebral vascular diseases.^4^, ^5^ Previous study has revealed that TXL can significantly reduce myocardial no‐reflow and infarction area after emergency percutaneous coronary intervention for acute ST‐segment elevation myocardial infarction.^6^ Our prior experimental study showed that TXL could alleviate cerebral microcirculatory disturbances in ischemic stroke in vivo observed by two‐photon microscopy and thus reduce brain infarct volume.^7^ Nonetheless, it is still not clear whether TXL can alleviate no‐reflow after vascular recanalization in ischemic stroke.

The underlying mechanisms of no‐reflow have not been fully delineated. Previous study has revealed that potential mechanisms of microvascular no‐reflow after stroke are multiple such as obstruction of blood‐borne elements (erythrocytes, leukocytes, platelets, and blood clots), compression of microvessels owing to swelling of endothelia and astrocyte end‐feet, and pericyte contraction of cerebral capillaries.^8^ Among different mechanisms, endothelia and leukocytes play a vital role in leading to no‐reflow after stroke. Thus, we propose that leukocyte‐endothelial cell interactions (adhesion, aggregation, and rolling) are a potential cause of no‐reflow after stroke. Moreover, it is revealed that adhesion molecules, and chemokines play a critical part in leukocyte‐endothelial cell interactions.^9^

Combined with our previous conclusion that TXL is capable of suppressing leukocyte‐endothelial cell interactions in ischemic stroke mice,^7^ we herein hypothesize that leukocyte‐endothelial cell interactions mediated by numerous adhesion molecules and chemokines are an important cause of no‐reflow after vascular recanalization in ischemic stroke, and TXL can alleviate the no‐reflow by inhibiting these biological targets. In this study, we used in vivo laser speckle cortical imaging to evaluate the effect of TXL on no‐reflow after vascular recanalization in ischemic stroke induced by transient middle cerebral artery occlusion (tMCAO), and further explored the potential mechanisms of no‐reflow and TXL's protective effects against the phenomenon from the perspective of regulating leukocyte‐endothelial cell interactions.

## 2 MATERIALS AND METHODS

### 2.1 Animals

All animal experiment protocols were approved by the Institutional Animal Ethics Committee of Dongzhimen Hospital, Beijing University of Chinese Medicine (Beijing, China; permit No. 22‐04) consistent with international guidelines for animal research. C57BL/6J male mice (6–8 weeks old, 22–24 g in weight) were purchased from Beijing HFK Biotech Co., Ltd (License: SCXK (Beijing) 2019‐0008). They were housed in SPF‐grade animal facility of Key Laboratory of Chinese Internal Medicine of Ministry of Education (Dongzhimen Hospital, Beijing University of Chinese Medicine) on a 12‐hour light/dark diurnal cycle with food and water ad libitum. A total of 157 animals were used in this study, including 118 mice for data analysis, 19 dead mice, and 16 excluded mice upon criteria below.

### 2.2 tMCAO model

Ischemic stroke with arterial recanalization was induced by transient middle cerebral artery occlusion (tMCAO) as previously described.^10^, ^11^ Brief surgical procedures of the model are as follows: mice were intraperitoneally anesthetized with 0.75% pentobarbital sodium (75 mg/kg).^12^ The left carotid arteries were isolated. Then a silicon‐coated monofilament suture (Guangzhou Jialing Biotechnology Co., Ltd) was inserted into the left common carotid artery and advanced along the left internal carotid artery until a slight resistance appeared (approximately 9–10 mm after the left carotid bifurcation). After 1.5 h of occlusion, reperfusion was performed through withdrawing the filament. Sham‐operated mice underwent identical procedures but without filament insertion. During surgery, animal body temperature was maintained at 37°C with a dependable electric blanket.

### 2.3 Drug preparation and administration

TXL, a Chinese medicine prescription, is clinically used in the form of capsules. It contains 12 components (Table 1) as previously described.^7^ These primary materials were ground to a superfine powder (≤10 μm) by the micronization technology and made into capsules standardized upon marker compounds according to the Chinese Pharmacopeia 11th edition.^13^ In this experiment, TXL superfine powder was employed rather than capsules. The powder was provided by Shijiazhuang Yiling Pharmaceutical Incorporated Company. In this study, the powder was dissolved in 0.9% sodium chloride (the suspension concentration 0.15 g/mL) and the suspension was stored at 4°C for no more than 3 days.

**TABLE 1:** Components of Tongxinluo capsule.

Components (Latin name)	Chinese name	Part used	Voucher specimen number	Processing	Amount used (%)
*Panax ginseng* C.A. Mey.	Ren shen	Root and rhizome	11,001	Extraction	1.677
*Hirudo nipponica* Whitman	Shui zhi	Dried body	12,004	Farina	27.330
*Buthus martensii* Karsch	Quan xie	Dried body	12,002	Farina	18.111
*Scolopendra subspinipes mutilans* L. Koch	Wu gong	Dried body	12,001	Farina	3.623
*Steleophage plancyi*	Tu biechong	Female dried body	12,003	Micro‐oryzae farina	18.111
*Cryptotympana pustulata* Fabricius	Chan tui	Skin	12,005	Farina	18.111
*Paeonia lactiflora* Pall.	Chi shao	Root	11,003	Extraction	1.558
*Santalum album* L.	Tan xiang	Heartwood of stem	11,004	Extraction	0.354
*Dalbergia odorifera* T. Chen	Jiang xiang	Heartwood of stem and root	11,005	Extraction	4.000
*Boswellia carteri* Birdw	Ru xiang	Resin	11,006	Farina	5.927
*Borneolum syntheticum*	Bing pian	Resin	11,007	Artificial	3.626
*Ziziphus jujube* Mill. Var. *spinosa* (Bunge) Hu H.F. Chou	Suan zaoren	Seed	11,002	Extraction	1.173

In the current study, we used the best therapeutic dose of TXL (3.0 g/kg) in reducing infarct volume and alleviating cerebral microcirculatory disturbances after stroke in three ones (0.75, 1.5, and 3.0 g/kg) according to our prior research.^7^ The tMCAO mice were excluded from the analysis in the following conditions at 1.5 h after tMCAO: no neurological deficit and consciousness disorder. Additionally, tMCAO mice with signs of intracranial hemorrhage confirmed during the collection of brains were also excluded. Mice were randomly divided into three groups: sham group (Sham), tMCAO group (tMCAO), and tMCAO+TXL group. The mice in tMCAO+TXL group were orally administered with TXL (3.0 g/kg) at 1.5 h, 22 h (2 h earlier than 24 h), 48 h, and 70 h (2 h earlier than 72 h) after stroke, while the other mice were done with the same volume normal saline at the corresponding time points.

### 2.4 Neurological assessment

All mice underwent neurological assessment at 6, 24 and 72 h after surgery in a blinded manner as previously described.^7^ Neurological scores were calculated at the sum of scores on motor function including hemiplegia (assessing via raising the mouse by the tail (score 0–3) and walking on the floor (score 0–3) and abnormal movement) (immobility and staring (score 0–1), tremor (score 0–1), and myodystonia, irritability or seizures (score 0–1), sensory function including tactile (score 0–1) and proprioceptive sensory (score 0–1), and reflexes including pinna reflex (score 0–1), corneal reflex (score 0–1), and startle reflex (score 0–1)). Neurological function was scored from 0 to 14 (normal score 0, maximal deficit score 14). The higher scores suggest more severe neurological deficit.

### 2.5 Laser speckle cortical imaging

The laser speckle imaging system (RFLSI III, Shenzhen RWD Life Science, Shenzhen, China) obtains high‐resolution and two‐dimensional imaging and has a linear relationship with absolute cerebral blood flow (CBF) values. Relative CBF values reflecting blood reperfusion can be determined by the imaging system. Thus, no‐reflow after stroke was assessed by laser speckle perfusion imaging as previously described.^1^ In brief, scalp of mice was open via a midline incision under 0.75% pentobarbital sodium anesthesia. Recordings of CBF in regions of interest (ROIs) were performed through the fixed skull using laser speckle perfusion imaging at 6, 24, and 72 h after stroke. For each recording, normal saline was added on the skull surface to prevent drying. Relative CBF was examined in identically sized ROIs of bilateral hemispheres. No‐reflow was assessed by CBF % of contralateral level.

### 2.6 Two‐photon microscopy imaging

Leukocyte imaging labeled by Rhodamine 6G (Sigma‐Aldrich) was performed by two‐photon microscopy (OLFV‐870SM/W7E‐1, Olympus, Japan) at 24 h or 72 h after stroke according to our prior method.^7^ Briefly, a cranial window of 3.5 mm in diameter was carried out on the top of the left parietal cortex (craniotomy window center: bregma 2.5 mm, left 2.5 mm) 15 min before in vivo imaging under anesthesia. The intact dura mater was reserved to protect cortex from adverse influence by external environment. A metal frame of diameter 7.0 mm of self‐made head fixation device was glued to the side of cranial window. Rhodamine 6G (0.04% in normal saline) was injected via the tail vein 2 minutes before in vivo imaging. Leukocytes were observed by two‐photon microscopy. The scanning mode was “time series” using resolution of 512 × 512 pixels. The relative number of adherent and aggregated leukocytes per cm^2^ area of venular walls was calculated with the software Image J (RRID: SCR_003070).

### 2.7 HE staining

Murine brains were immediately collected at 24 h and 72 h after stroke. Then they were fixed 4% paraformaldehyde for more than 24 h and embedded in paraffin. Coronal sections of 5 μm in thickness were prepared, stained with hematoxylin and eosin (HE) and subsequently imaged using an optical microscope.

### 2.8 Flow cytometry analysis of leucocytes in peripheral blood

Leukocyte subpopulations in peripheral blood were analyzed by flow cytometry as previously described.^14^, ^15^, ^16^, ^17^, ^18^ In brief, 500 μL blood from the vessels behind eyeballs was collected in a tube with EDTA anticoagulant. Leukocytes were stained with the following fluorescently labeled antibodies (BD Pharmingen™): APC‐Cy™7 Rat Anti‐Mouse CD45 (Cat. 557,659), BV605 Rat Anti‐Mouse LY‐6G (Cat. 563,005), FITC Rat Anti‐CD11b (Cat. 557,396), BV421 Rat Anti‐Mouse F4/80 (Cat. 565,411), PE Rat Anti‐Mouse CD86 (Cat. 561,963), Alexa Fluor® 647 Rat Anti‐Mouse CD206 (Cat. 565,250), PE‐Cy™7 Hamster Anti‐Mouse CD11c (Cat. 561,022), BV786 Rat Anti‐Mouse CD45R/B220 (Cat. 563,894), BV650 Hamster Anti‐Mouse CD3e (Cat. 564,378), PE‐Cy™7 Rat Anti‐Mouse CD4 (Cat. 561,099), BV510 Rat Anti‐Mouse CD8a (Cat. 563,068), BB515 Rat Anti‐Mouse CD25 (Cat. 564,458), BV421 Rat Anti‐Mouse CD127 (Cat. 566,300), PE Mouse Anti‐Mouse NK‐1.1 (Cat. 561,046), APC Rat Anti‐Mouse IFN‐γ (Cat. 562,018), PE Rat Anti‐Mouse IL‐4 (Cat. 562,044) and BV421 Rat Anti‐Mouse IL‐17A (Cat. 566,286). After blood cells were incubated with fluorescent antibodies above for 30 min at 4°C away from light, red blood cells were lysed by lysing buffer (BD Pharm Lyse™, Cat. 555,899) for 15 min at room temperature. Samples were centrifuged at a speed of 300 *g* for 5 min, and the supernatant was discarded. Remaining cells were washed twice with PBS, and were resuspended in 400 μL PBS. Then samples were determined by flow cytometry (Beckman DeFLEX). For detecting Th1, Th2, and Th17, the following steps were also included: samples were activated by leukocyte activation cocktail (BD GolgiPlug™, Cat. 550,583) at 37°C and 5% CO_2_, and then fixed and permeabilized for 20 min at 4°C away from light using the fixation/permeabilization kit (BD Cytofix/Cytoperm™, Cat. 554,714). After the supernatant was discarded, remaining cells were washed and subsequently stained with APC Rat Anti‐Mouse IFN‐γ, PE Rat Anti‐Mouse IL‐4, and BV421 Rat Anti‐Mouse IL‐17A. Then samples were determined by flow cytometry after being washed and resuspended. Data were analyzed with Kaluza Analysis (Beckman).

### 2.9 Cytometric bead array for measurement of cytokines

Ischemic brain and blood were collected at 24 h and 72 h after stroke. Brain tissue was ground and then was filtered by a cell strainer of diameter 40 μm. Plasma was separated by centrifugation at a speed of 3500 r/min for 8 min. Processed brain and plasma were stored at −80°C until further analysis by cytometric bead array (CBA). CBA was performed for examining adhesion molecules (P‐selectin, E‐selectin, ICAM‐1, and VCAM‐1) and chemokines (CCL‐2, CCL‐3, CCL‐4, CCL‐5, and CXCL1) according to the protocols of manufacturer (Antigenix America).

### 2.10 Statistical analysis

Statistical analysis was conducted with SPSS 16.0 (RRID:SCR_002865) in a blinded manner that analyzers were and are unaware of the experimental details. All data distribution was assessed by the test of homogeneity of variance. Data meeting the homogeneity of variance were analyzed by using one‐way ANOVA. The test was employed to detect the significance of differences between multiple comparisons, and LSD test was used between two arbitrary comparisons as a post hoc test. For some data that did not meet the homogeneity of variance, nonparametric test (Kruskal‐Wallis) be applied to analyze them. Significance was set at *p* < 0.05 or *p* < 0.01. All quantitative results were expressed as means ± standard deviation (SD).

## 3 RESULTS

### 3.1 TXL alleviated neurological deficits after tMCAO

**FIGURE 1:**
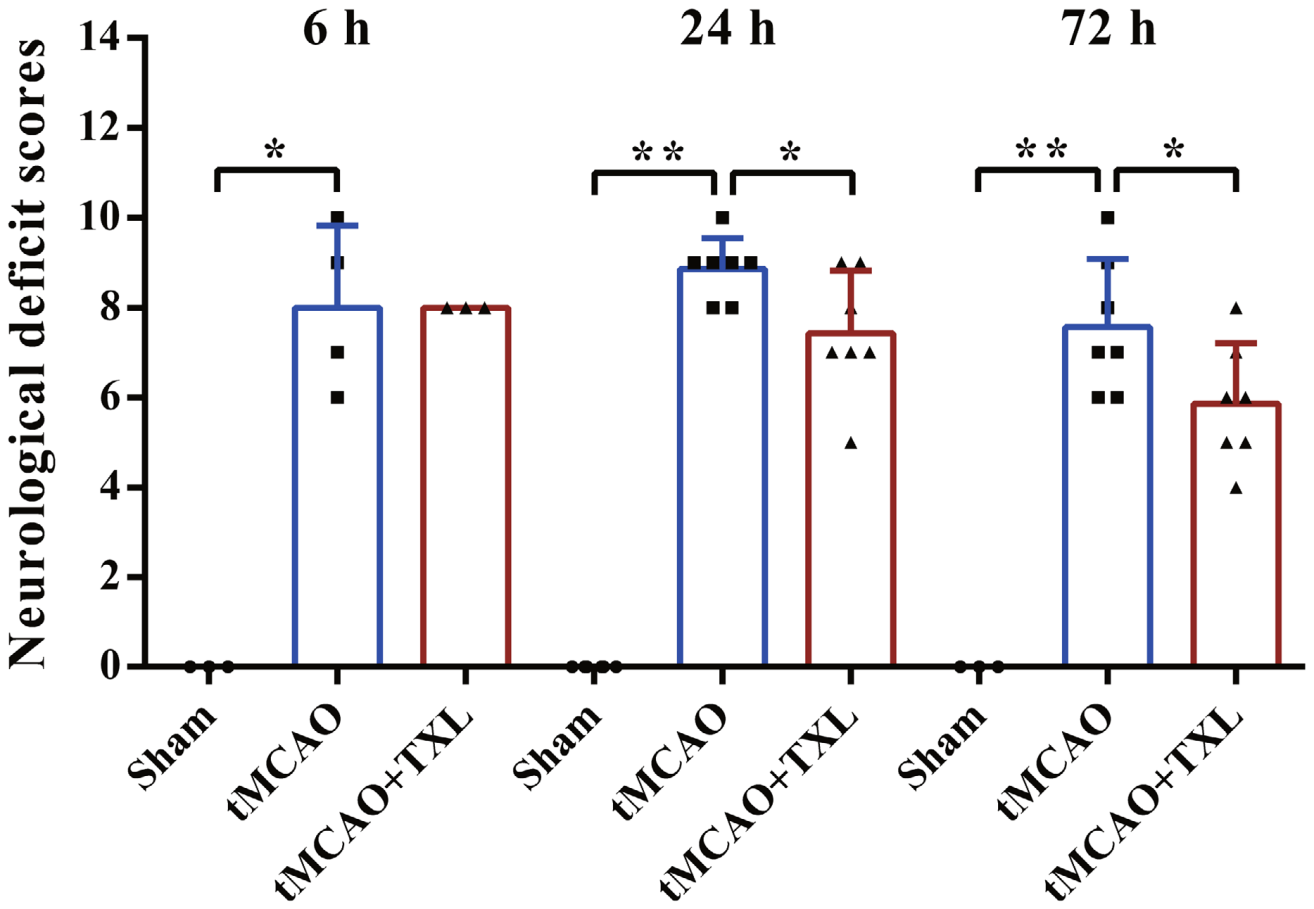
TXL alleviated neurological deficits after tMCAO. Neurological deficits in different groups at 6, 24, and 72 h. Data were expressed as means ± SD. *N* = 3–7 mice each group. *p < 0.05, **p < 0.01.

Neurological function scores were performed at three time points (6, 24, and 72 h after stroke). Our results (Figure 1) showed that the scores in tMCAO group were significantly higher than these in sham group at these three time points indicating that the stroke model was successful. After treatment with TXL, the scores were reduced compared with these in tMCAO group at 24 h or 72 h. At 6 h, there was no difference in scores between tMCAO+TXL group and tMCAO group. These results revealed that TXL could alleviate neurological deficits after ischemic stroke.

### 3.2 TXL improved no‐reflow after arterial recanalization in ischemic stroke

**FIGURE 2:**
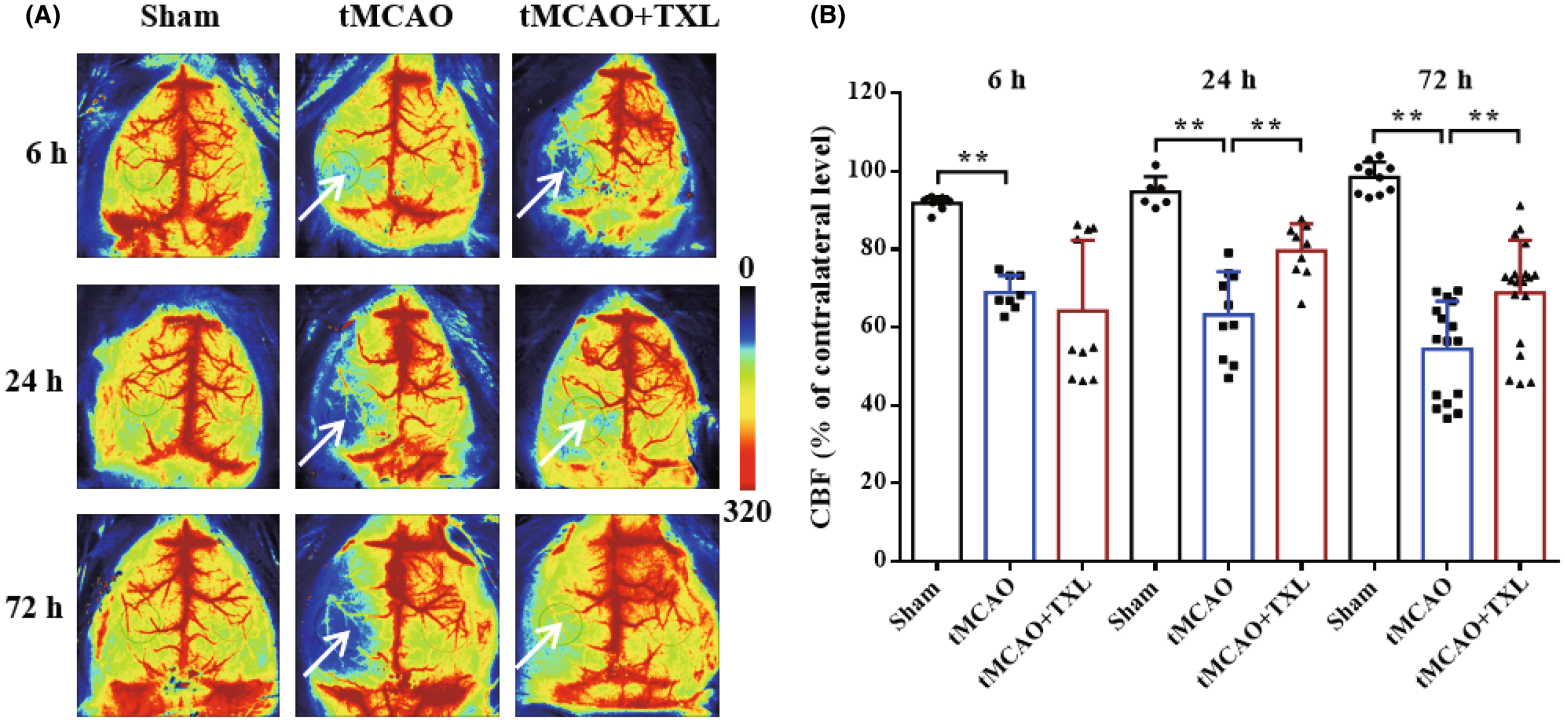
TXL improved no‐reflow after arterial recanalization in ischemic stroke. (A) Representative CBF images by laser speckle perfusion imaging in different groups at 6, 24, and 72 h. Circles indicate regions of interest showing blood‐supplying zones of left middle cerebral artery. Arrows suggest suppression of no‐reflow in ischemic stroke after treatment of TXL. (B) Quantitative analysis of CBF in the respective ROIs compared to contralateral level in the same zones at 6, 24, and 72 h. *N* = 3–4 mice each group, *n* = 2–7 measurements each mouse. **p < 0.01.

No‐reflow was evaluated by laser speckle perfusion imaging. At 1.5 h after brain ischemia, vascular recanalization was induced by removing the monofilament. However, after vascular recanalization, CBF recovered to around 60% of contralateral cerebral cortex indicative of no‐reflow phenomenon. After treatment of TXL, CBF was significantly increased at 24 h or 72 h compared with that in tMCAO mice. At 6 h, there was no difference in CBF between tMCAO+TXL group and tMCAO group. These results (Figure 2) suggested that no‐reflow did occur after vascular recanalization in ischemic stroke, and TXL could alleviate the phenomenon.

### 3.3 TXL ameliorated leukocyte‐endothelial cell interactions after tMCAO

**FIGURE 3:**
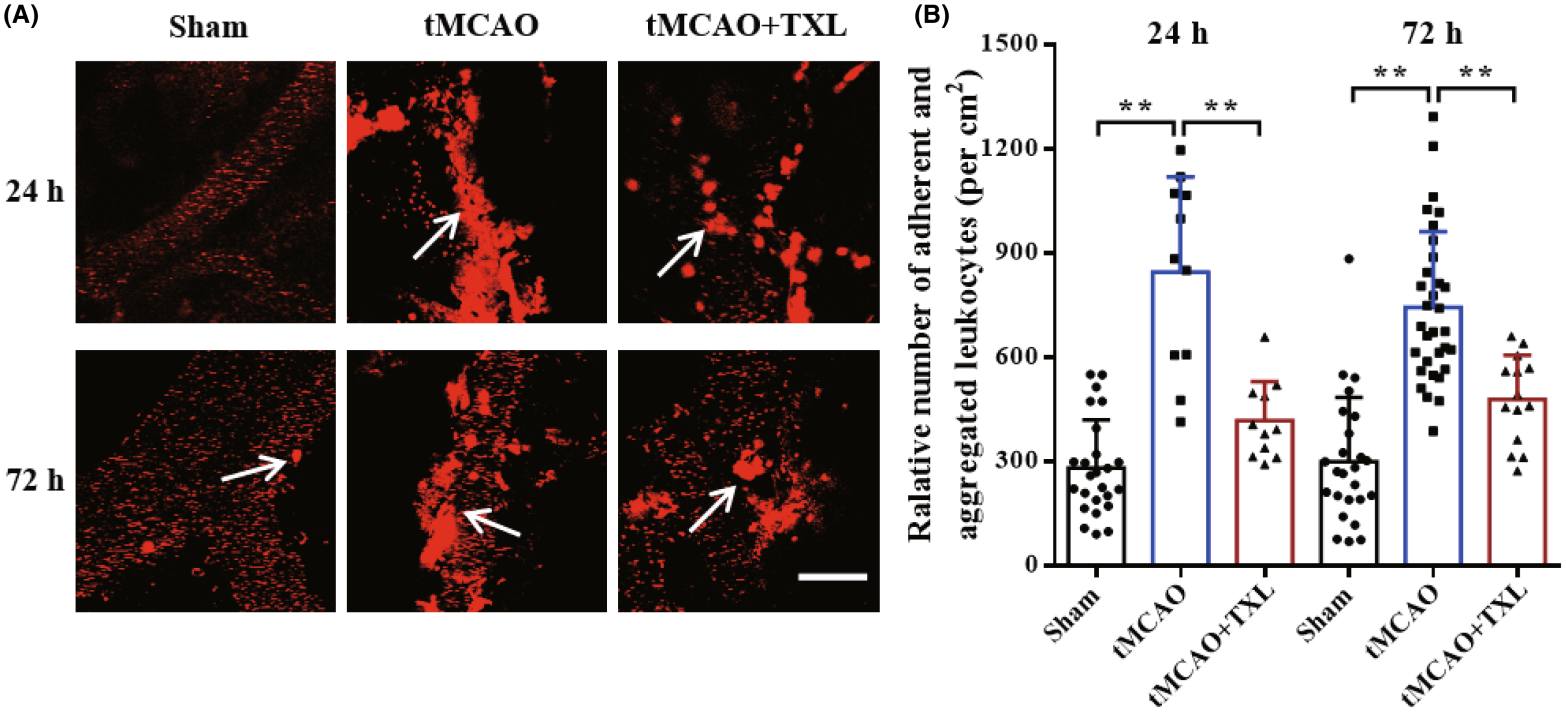
TXL ameliorated leukocyte‐endothelial cell interactions after tMCAO. Two‐photon imaging of leukocytes labeled by Rhodamine 6G in cortex microvessels at 24 h and 72 h. (A) Representative images of leukocyte‐endothelial cell interactions (aggregation and adhesion) directed by the white arrows, and (B) quantitative data of relative number of adherent and aggregated leukocytes per cm^2^ in cortex venules. *N* = 3–4 mice each group, *n* = 1–12 measurements of leukocytes each mouse. **p < 0.01. Scale bar = 50 μm.

Leukocyte‐endothelial cell interactions were examined by two‐photon microscopy. Our results (Figure 3) showed that there were only a few adherent and aggregated leukocytes in sham group at 24 h or 72 h. After stroke, adherent and aggregated leukocytes were markedly increased, and it was in vivo clearly observed that these leukocytes obstructed cerebral microvessels and then impaired blood reperfusion (no‐reflow). However, upon treatment of TXL, leukocyte‐endothelial cell interactions were significantly inhibited, and then the obstruction of microvessels was alleviated. These results suggested that adherent and aggregated leukocytes obstruct brain microvessels and are an important cause of no‐reflow after arterial recanalization in ischemic stroke, and TXL could ameliorate leukocyte‐endothelial cell interactions and subsequent no‐reflow in stroke.

### 3.4 TXL reduced leukocyte infiltration and protected neurons after tMCAO

**FIGURE 4:**
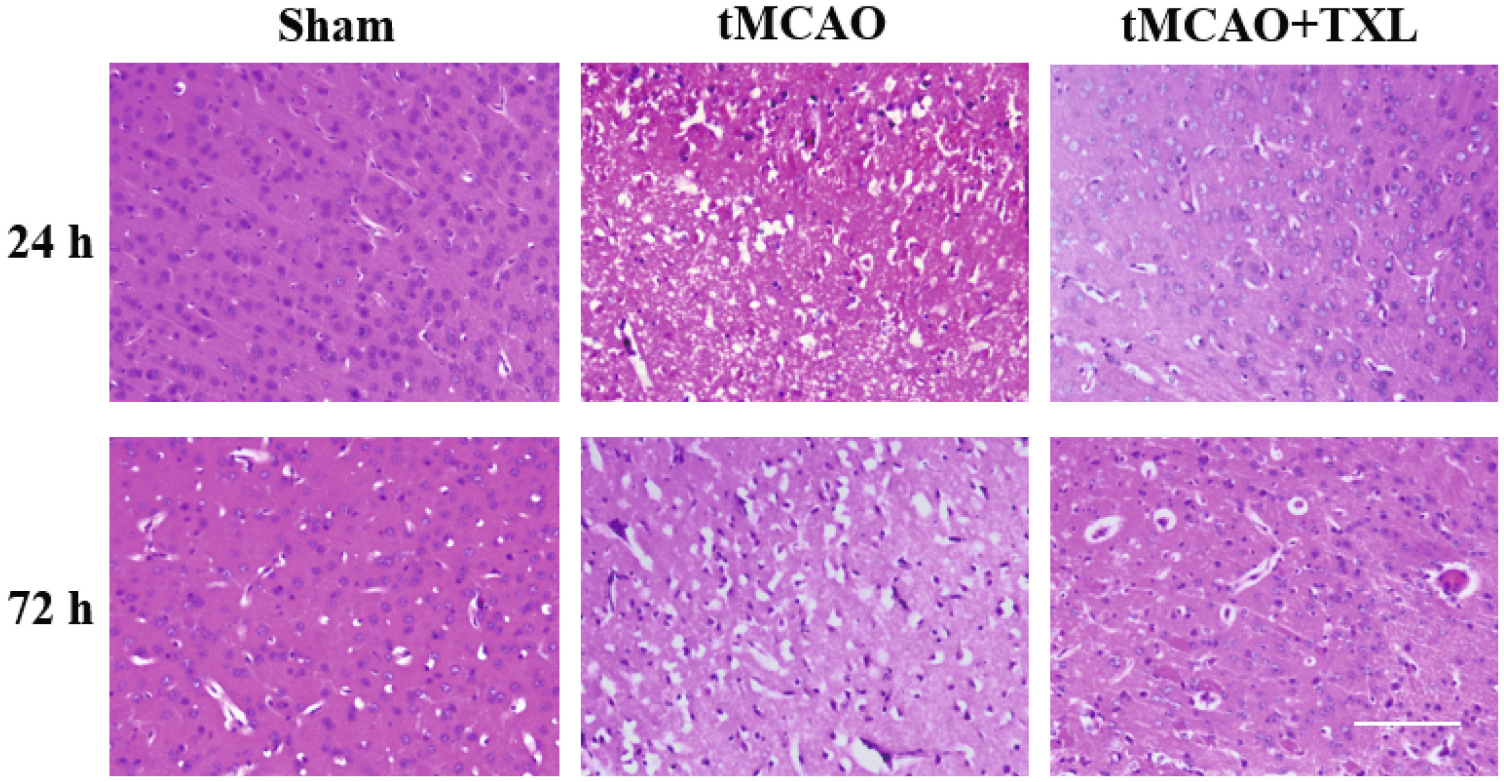
TXL reduced leukocyte infiltration and protected neurons after tMCAO. HE‐stained sections of ischemic brain at 24 h and 72 h after tMCAO. *N* = 3 mice each group. Scale bar = 100 μm.

To further study the protective effect of TXL on neurons in ischemic brain regions, inflammatory cell infiltration and neuron death were observed by HE staining at 24 h and 72 h. The tMCAO group showed a larger amount of inflammatory cell infiltration and neuron death than those of the sham group at 24 h and 72 h. Treatment with TXL ameliorated the pathological severity (Figure 4).

### 3.5 TXL regulated distribution of different immune cells in peripheral blood after tMCAO

**FIGURE 5:**
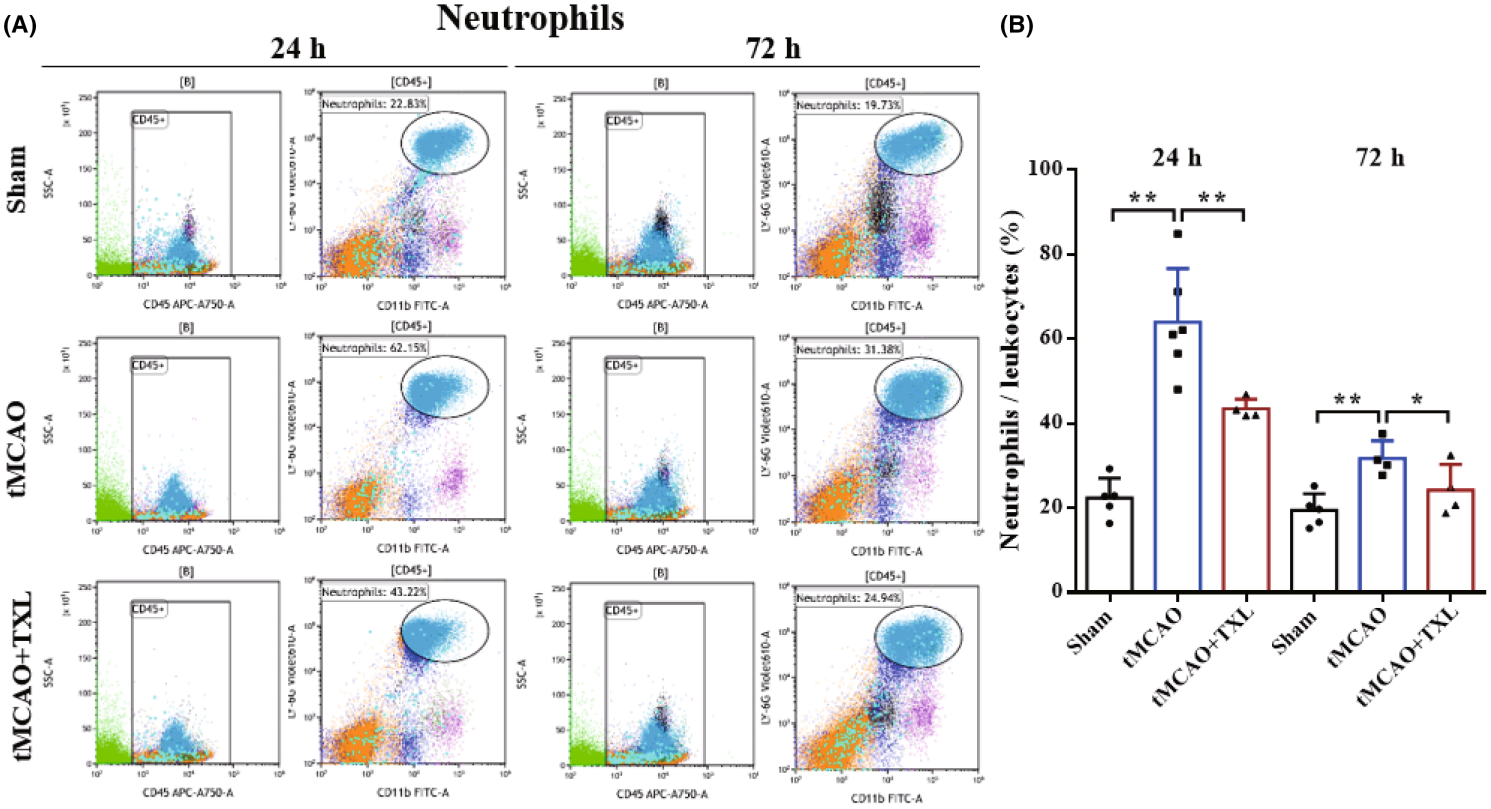
TXL reduced the ratio of neutrophils in peripheral blood after tMCAO. Representative flow cytometry dot plots and quantitative analysis for neutrophils (CD45^+^ly6G^+^CD11b^+^) among different groups at 24 h and 72 h after tMCAO. *N* = 4–6 mice each group. *p < 0.05 and **p < 0.01.

**FIGURE 6:**
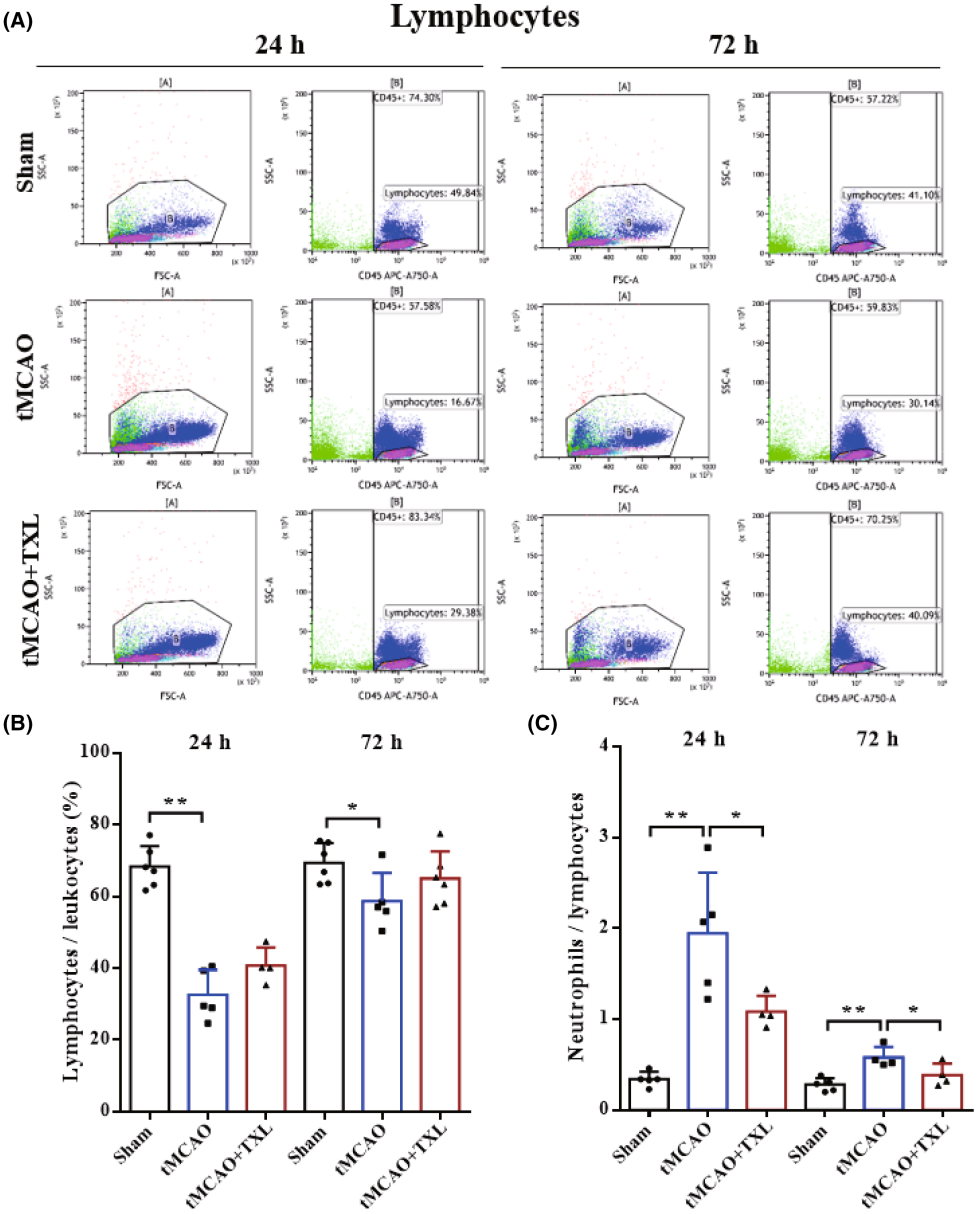
Effect of TXL on lymphocytes and N/L in peripheral blood after tMCAO. Representative flow cytometry dot plots and quantitative analysis for lymphocytes and N/L among different groups at 24 h and 72 h after tMCAO. *N* = 4–6 mice each group. *p < 0.05 and **p < 0.01.

**FIGURE 7:**
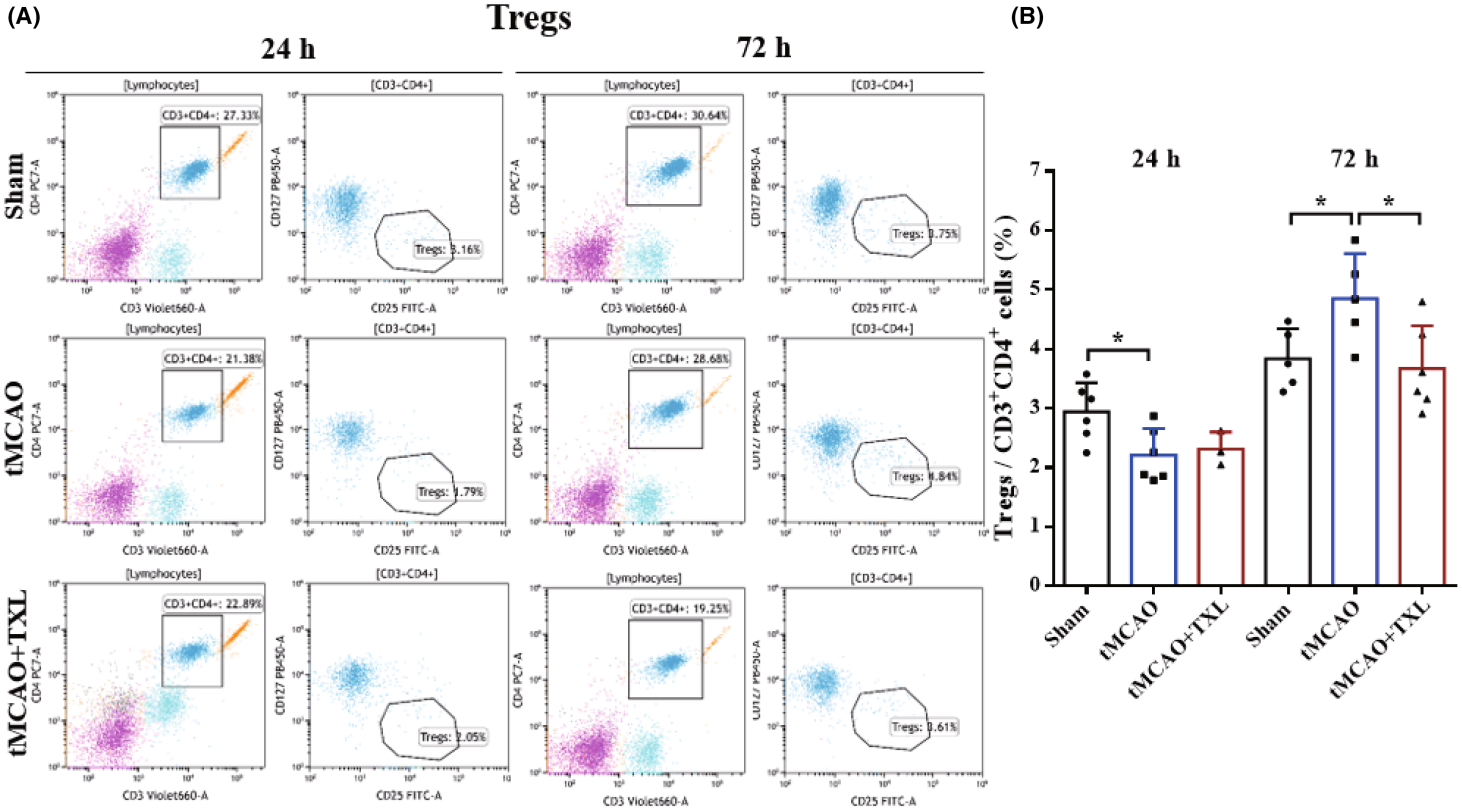
TXL reduced the ratio of Tregs in peripheral blood after tMCAO. Representative flow cytometry dot plots and quantitative analysis for Tregs (CD4^+^CD25^+^CD127^−^) among different groups at 24 h and 72 h after tMCAO. *N* = 4–6 mice each group. *p < 0.05.

**FIGURE 8:**
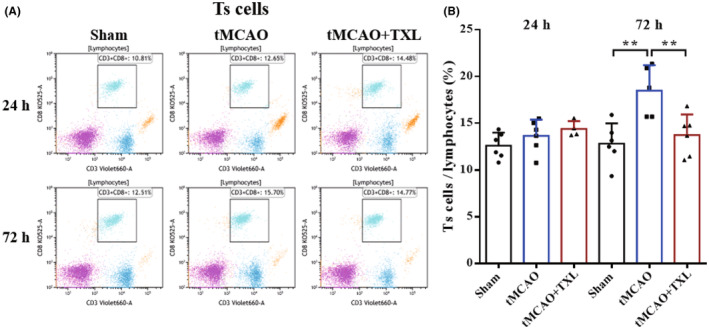
TXL reduced the ratio of Ts cells in peripheral blood after tMCAO. Representative flow cytometry dot plots and quantitative analysis for Ts cells (CD3^+^CD8^+^) among different groups at 24 h and 72 h after tMCAO. *N* = 4–6 mice each group. **p < 0.01.

**FIGURE 9:**
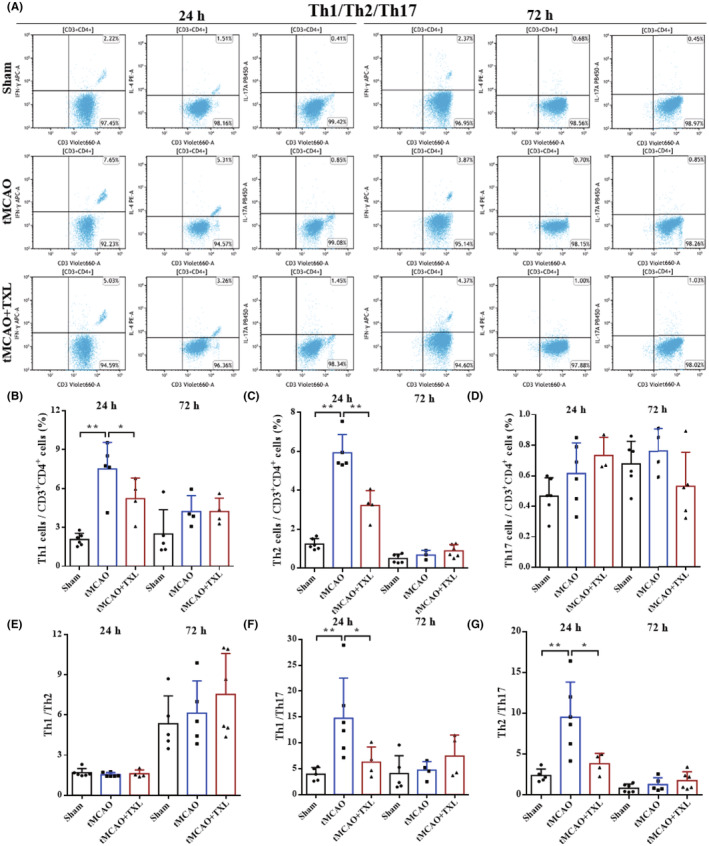
Effect of TXL on Th cells in peripheral blood after tMCAO. Representative flow cytometry dot plots (A) and quantitative analysis for Th1 (CD3^+^CD4^+^IFN‐γ^+^) (B), Th2 (CD3^+^CD4^+^IL‐4^+^) (C), Th17 cells (CD3^+^CD4^+^IL‐17A^+^) (D), Th1/Th2 (E), Th1/Th17 (F) and Th2/Th17 (G) among different groups at 24 h and 72 h after tMCAO. *N* = 4–6 mice each group. *p < 0.05 and **p < 0.01.

**FIGURE 10:**
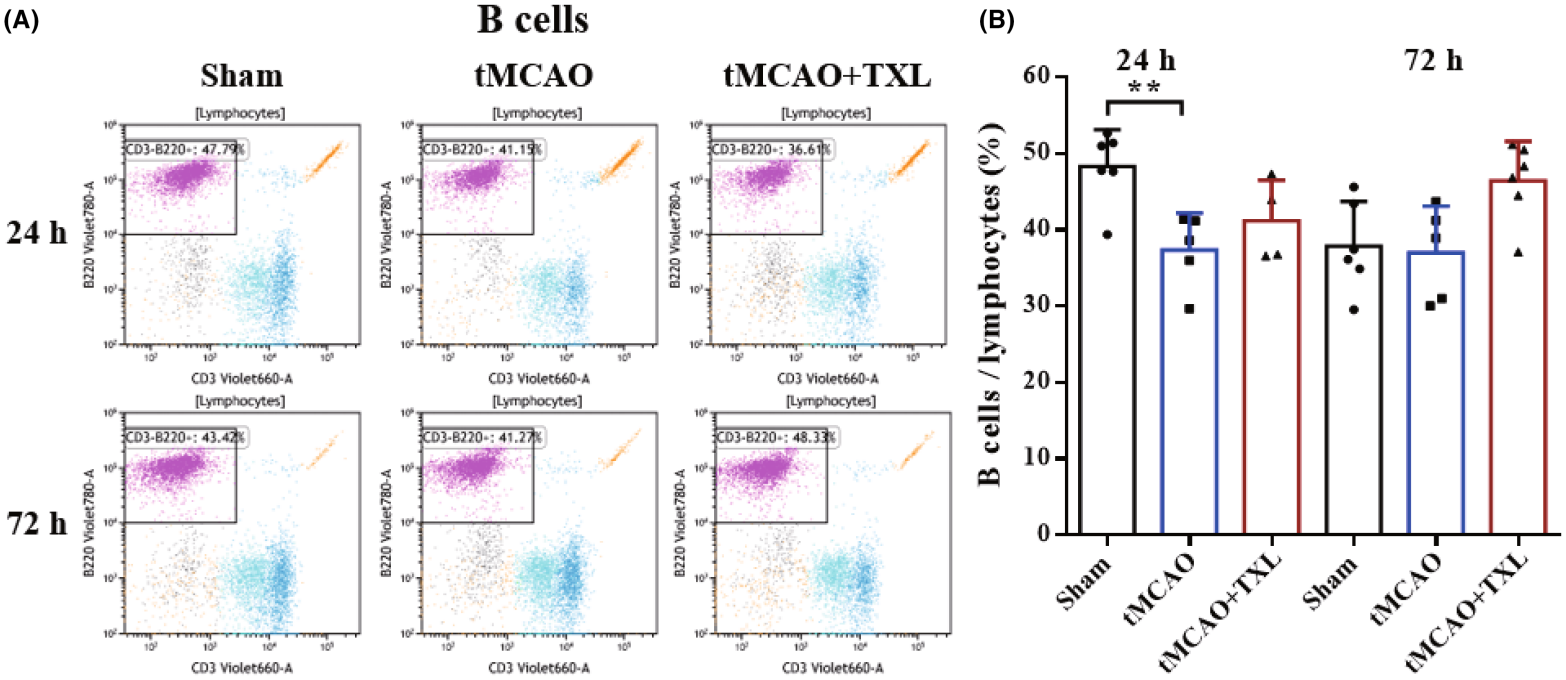
Effect of TXL on B cells in peripheral blood after tMCAO. Representative flow cytometry dot plots (A) and quantitative analysis for B cells (CD3^−^B220^+^) (B) among different groups at 24 h and 72 h after tMCAO. *N* = 4–6 mice each group. **p < 0.01.

**FIGURE 11:**
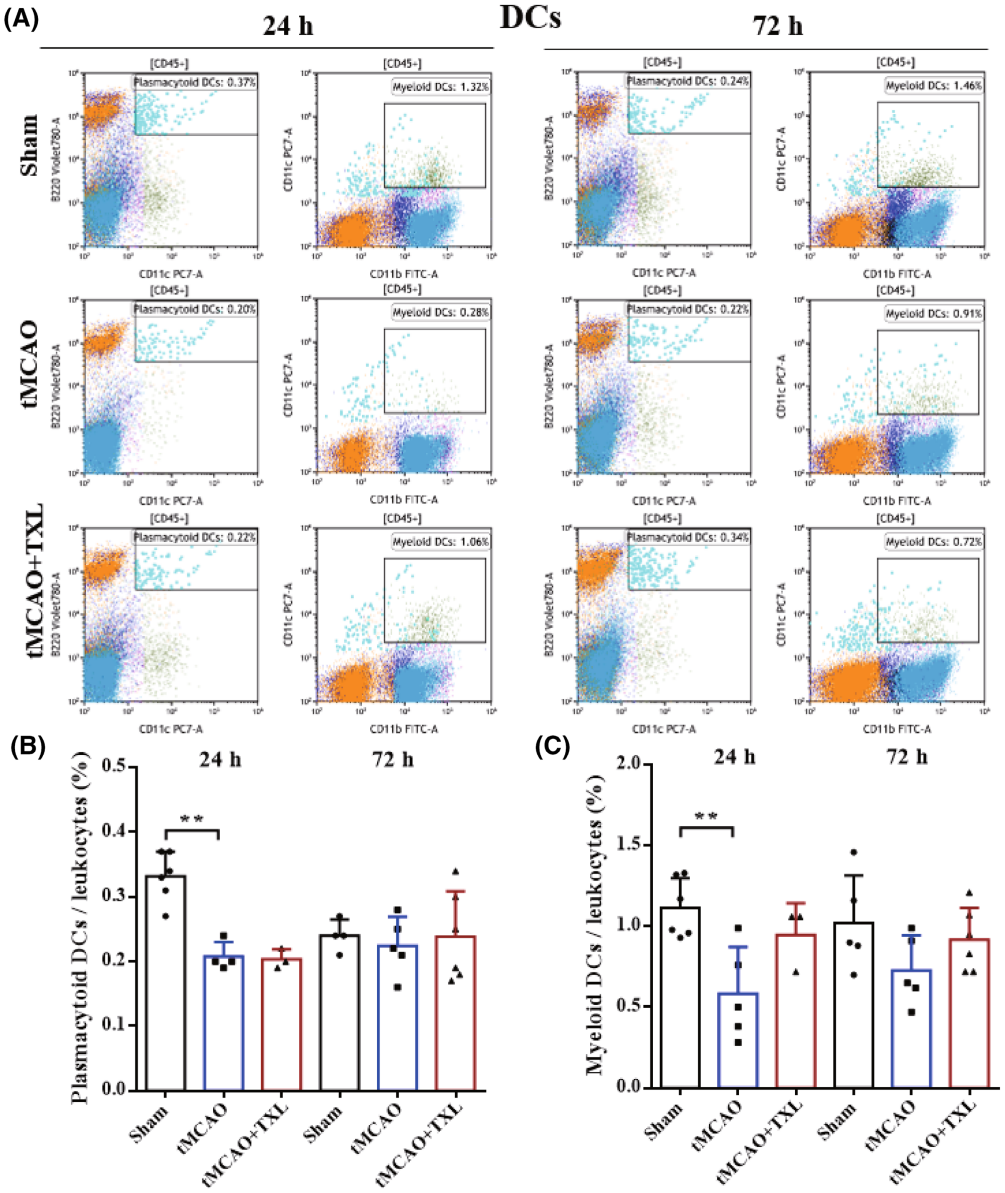
Effect of TXL on DCs in peripheral blood after tMCAO. Representative flow cytometry dot plots (A) and quantitative analysis for plasmacytoid DCs (CD11c^+^B220^+^) (B) and myeloid DCs (CD11c^+^ CD11b^+^) (C) among different groups at 24 h and 72 h after tMCAO. *N* = 4–6 mice each group. **p < 0.01.

**FIGURE 12:**
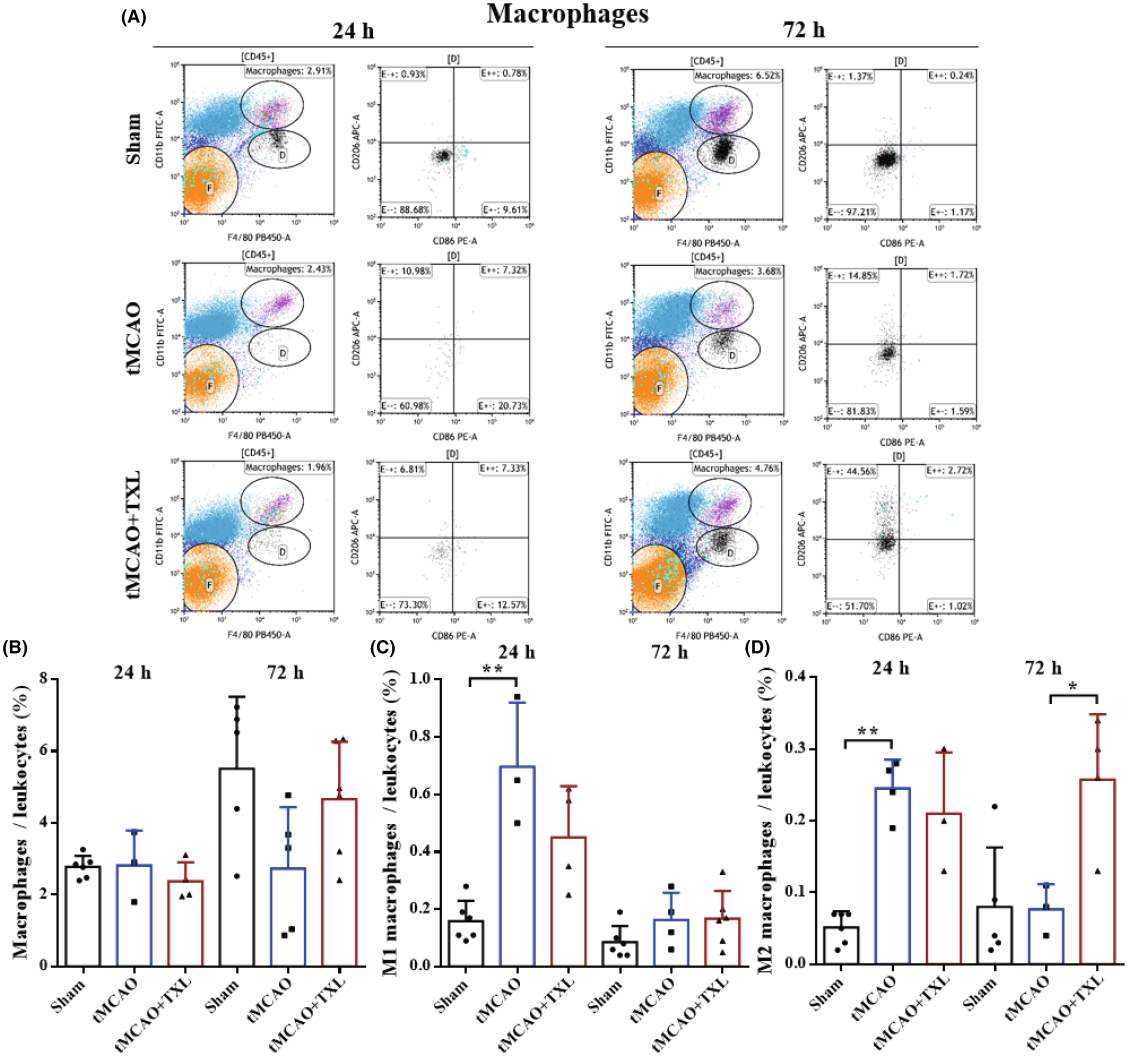
Effect of TXL on macrophages in peripheral blood after tMCAO. Representative flow cytometry dot plots (A) and quantitative analysis for macrophages (CD11b^+^F4/80^+^) (B), M1 macrophages (CD11b^+^F4/80^+^CD86^+^) (C), and M2 macrophages (CD11b^+^F4/80^+^CD206^+^) (D) among different groups at 24 h and 72 h after tMCAO. *N* = 4–6 mice each group. *p < 0.05 and **p < 0.01.

**FIGURE 13:**
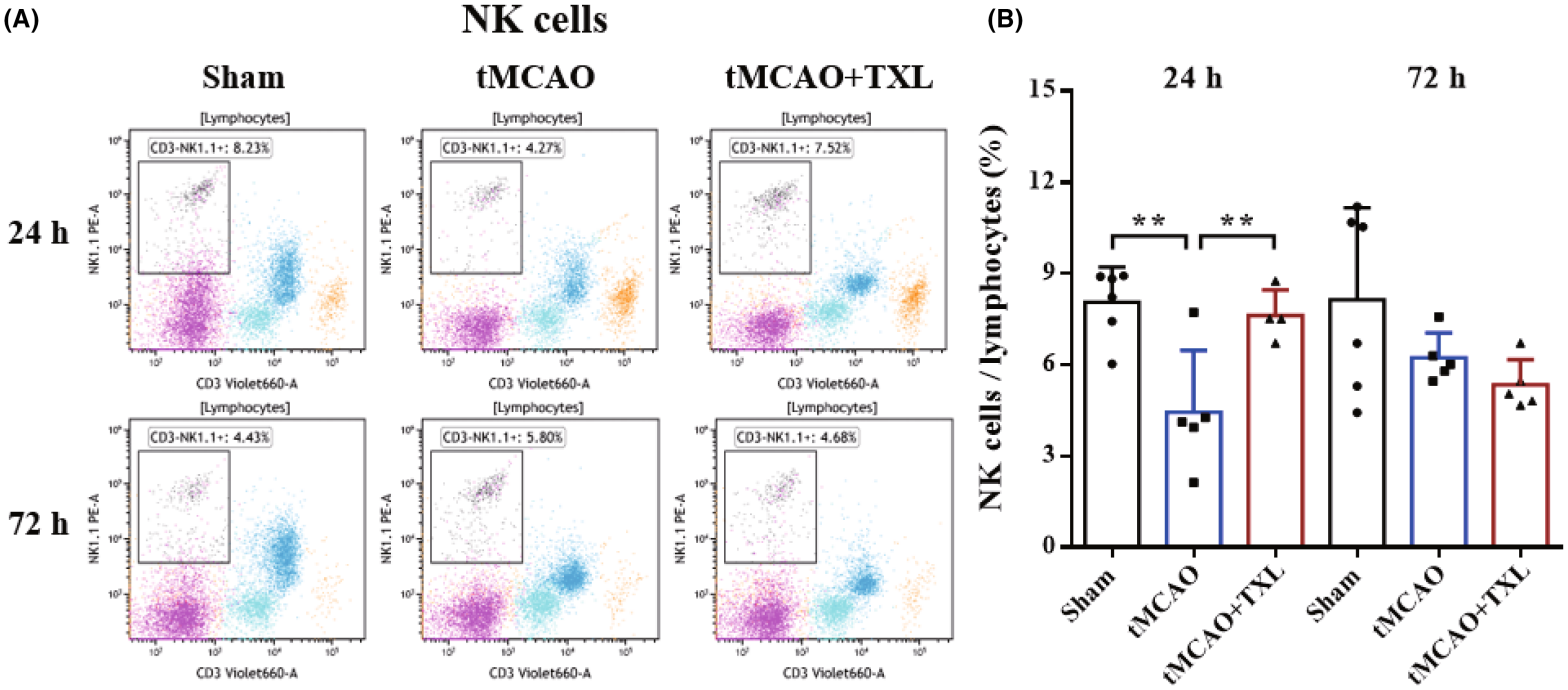
Effect of TXL on NK cells in peripheral blood after tMCAO. Representative flow cytometry dot plots (A) and quantitative analysis for NK cells (CD3^−^NK1.1^+^) (B) among different groups at 24 h and 72 h after tMCAO. *N* = 4–6 mice each group. **p < 0.01.

Different immune cells in peripheral blood after tMCAO at 24 h and 72 h were examined by flow cytometry. Our results showed that both percentages of neutrophils (Figure 5) and ratio of neutrophils to lymphocytes (N/L) (Figure 6C) were significantly higher in tMCAO group than those in sham group at 24 h or 72 h. After treatment of TXL, neutrophil percentage and N/L were reduced compared with those in tMCAO group at the two time points. Lymphocyte percentage (Figure 6A,B) was significantly less in tMCAO group than that in sham group at 24 h or 72 h. In contrast, there were no differences in lymphocyte percentage between tMCAO group and tMCAO+TXL group at the two time points. Both of regulatory T lymphocyte (Treg) (Figure 7) and suppressor T (Ts) cell (Figure 8) percentages were increased in tMCAO group compared with those in sham group at 72 h. After treatment of TXL, the two parameters were reduced compared with those in tMCAO group at 72 h. Interestingly, Treg percentage was decreased after tMCAO at 24 h. Whereas, there were no differences in Tregs between tMCAO+TXL group and tMCAO group, and in Ts cells among the three groups at 24 h. All of helper T type 1 (Th1) and Th2 cell percentages, and Th1/Th17 and Th2/Th17 ratio were increased in tMCAO group compared with those in sham group at 24 h. After treatment of TXL, these four parameters were reduced compared with those in tMCAO group at 24 h. However, there were no differences among the three groups in Th1 and Th2 percentages, Th1/Th17, and Th2/Th17 at 72 h, and in Th17 percentage and Th1/Th2 at 24 h and 72 h (Figure 9). At 24 h, percentages of B cells (Figure 10) and dendritics cells (DCs) (Figure 11) were decreased after tMCAO. Nonetheless, there were no differences in the two parameters between tMCAO+TXL group and tMCAO group at 24 h, and among the three groups at 72 h. There were no differences among the three groups in macrophages at the two time points. Interestingly, percentages of M1 and M2 macrophages were increased after tMCAO only at 24 h. However, TXL did not influence them at 24 h and conversely increased M2 macrophages at 72 h (Figure 12). The ratio of NK cells to lymphocytes was decreased compared with that in sham group. Nevertheless, TXL increased the ratio of NK cells when compared with that in tMCAO group (Figure 13). In summary, ischemic stroke led to leukocyte subtype changes in peripheral blood mainly including neutrophils, lymphocytes, N/L, Tregs, Ts cells, Th1, Th2, Th1/Th17, Th2/Th17, B cells, DCs, macrophages, and NK cells. Interestingly, most of these leukocyte subtype changes were exerted in a time‐dependent manner such as Tregs, Ts cells, Th1/Th17, Th2/Th17, B cells, DCs, macrophages, and NK cells. Accordingly, TXL could downregulate percentages of neutrophils, Tregs, Ts cells, Th1, Th2, N/L, Th1/Th17, and Th2/Th17, and upregulate NK cell percentage after tMCAO.

### 3.6 TXL suppressed the expression of adhesion molecules and chemokines after tMCAO

**FIGURE 14:**
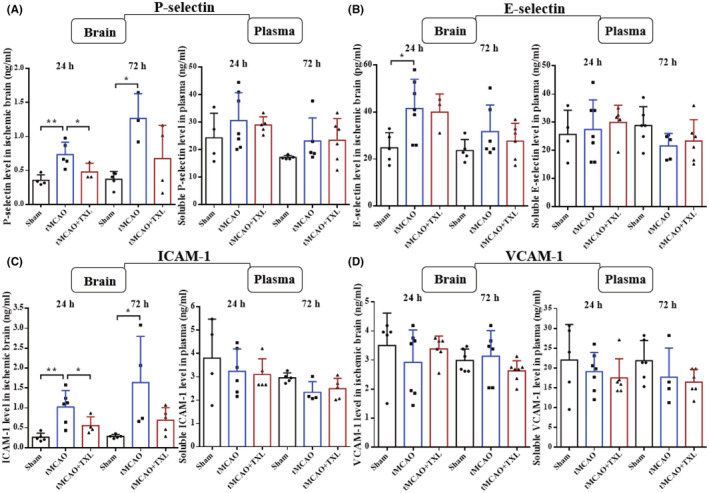
Effect of TXL on the expression of adhesion molecules after tMCAO. CBA analysis for adhesion molecules (P‐selectin (A), E‐selectin (B), ICAM‐1 (C) and VCAM‐1 (D)) in ischemic brain and plasma at 24 h and 72 h after tMCAO. *N* = 6–7 mice each group. *p < 0.05 and **p < 0.01.

**FIGURE 15:**
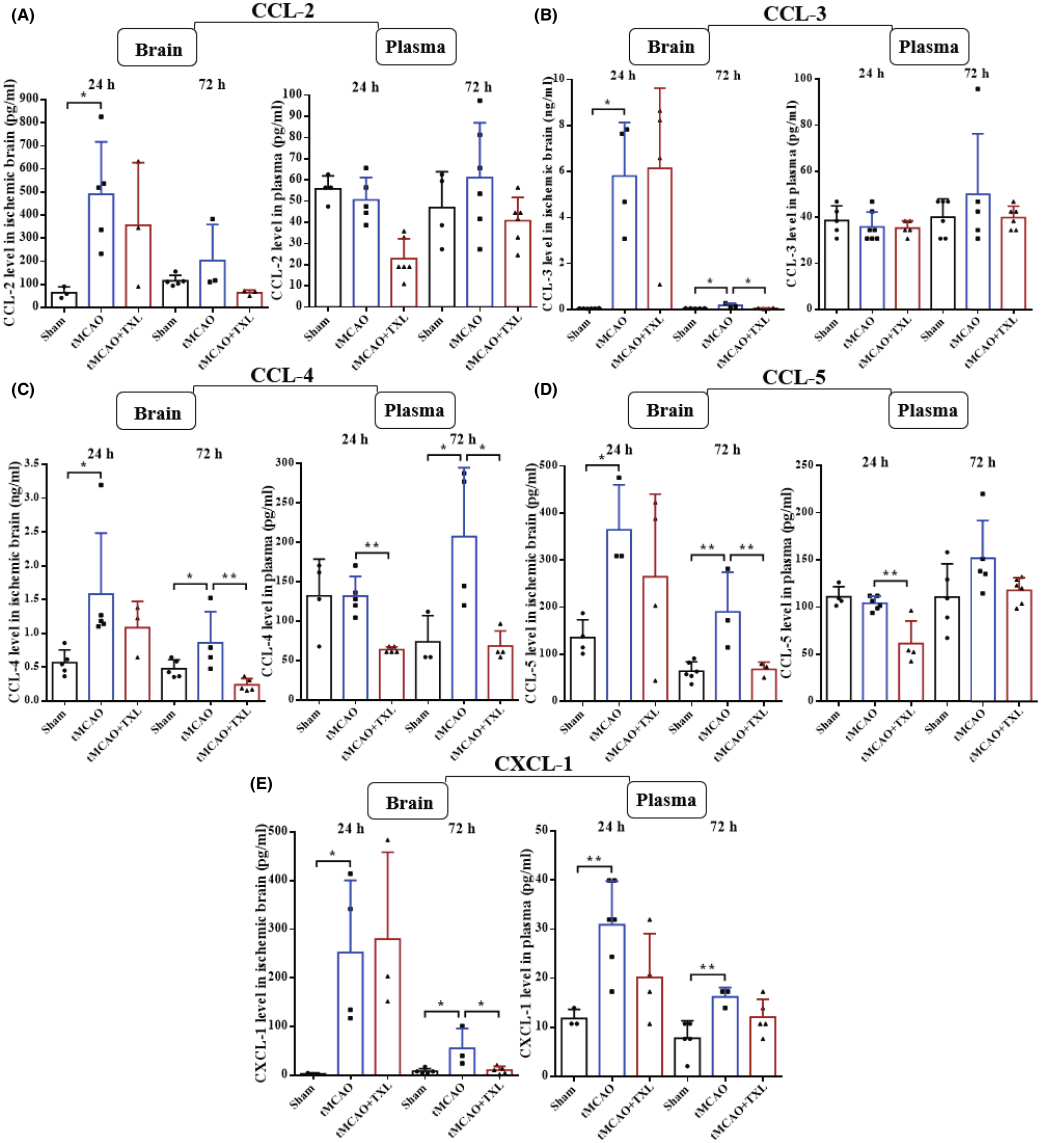
Effect of TXL on the expression of chemokines after tMCAO. CBA analysis for chemokines (CCL‐2 (A), CCL‐3 (B), CCL‐4 (C), CCL‐5 (D) and CXCL‐1 (E)) in ischemic brain and plasma at 24 h and 72 h after tMCAO. *N* = 6–7 mice each group. *p < 0.05 and **p < 0.01.

Adhesion molecules and chemokines in plasma and brain were measured with CBA at 24 h and 72 h after tMCAO. The levels of P‐selectin (Figure 14A), E‐selectin (Figure 14B) and CCL‐2 (Figure 15A) at 24 h, CXCL‐1 (Figure 15E) at 72 h and ICAM‐1 (Figure 14C), CCL‐3 (Figure 15B), CCL‐4 (Figure 15C) and CCL‐5 (Figure 15D) at 24 h and 72 h in ischemic brain of tMCAO group were apparently higher than those in sham group at 24 h or 72 h. In plasma, the expression of only CCL‐4 at 72 h and CXCL‐1 at 24 h in tMCAO group was increased when compared with that in sham group. After treatment of TXL, the levels of P‐selectin, and ICAM‐1 at 24 h, CCL‐3, CCL‐4, CCL‐5, and CXCL‐1 at 72 h in ischemic brain, and CCL‐4 at 24 h and 72 h and CCL‐5 at 24 h in plasma were reduced when compared with those in tMCAO group at 24 h or 72 h. There were no statistical differences in other conditions. In summary, ischemic stroke results in upregulated expression of adhesion molecules (P‐selectin, E‐selectin, and ICAM‐1) and chemokines (CCL‐2, CCL‐3, CCL‐4, CCL‐5, and CXCL‐1). Impressively, TXL could inhibit the stroke‐enhanced expression of adhesion molecules (P‐selectin and ICAM‐1) and chemokines (CCL‐3, CCL‐4, CCL‐5, and CXCL1).

## 4 DISCUSSION

Successful vascular recanalization strategies for ischemic stroke patients have evolved over the last decades. However, arterial recanalization after stroke does not equate with successful tissue reperfusion. Part of the recanalization is futile due to microvascular no‐reflow.^3^ The phenomenon after vascular recanalization in stroke has been a challenging bottleneck of successful tissue reperfusion and functional recovery from stroke. In the present study, we have verified that no‐reflow occurs after vascular recanalization in ischemic stroke using a mouse tMCAO model by laser speckle cortical imaging. Effective strategies to counteract no‐reflow after stroke do not exist in western medicine. In this regard, unblocking‐collateral therapy based on the collateral disease theory of TCM is emerging to confer a potential strategy for tackling no‐reflow after stroke.

TXL is a representative Chinese medicine based upon unblocking‐collateral therapy. It has been used to clinically treat angina pectoris and ischemic cerebrovascular diseases since being approved by the State Food and Drug Administration of China in 1996.^19^ Our prior study showed that TXL could reduce cerebral infarct volume and improve neurological function after brain ischemia in mice.^7^ According to the collateral disease theory of TCM, TXL can unblock obstructed collaterals. We have experimentally confirmed that TXL could alleviate cerebral microcirculatory disturbances after ischemic stroke observed by two‐photon microscopy and protect brain microvessels after brain ischemia in mice.^7^ It is known that brain microvascular obstruction is the essence of no‐reflow after stroke. In addition, TXL could reduce myocardial no‐reflow in myocardial infarction mentioned above.^6^ Therefore, we postulated TXL could inhibit no‐reflow after stroke. In the present study, we have firstly confirmed this hypothesis using the tMCAO model clinically mimicking ischemic stroke with arterial recanalization.

Subsequently, we explored the mechanisms of no‐reflow after ischemic stroke and TXL suppressing the phenomenon. Ames and coworkers initially found brain no‐reflow phenomenon in stroke model in 1968.^20^ Its potential mechanisms are multiple as mentioned above. Among different mechanisms, endothelia and leukocytes play a vital role in leading to no‐reflow after stroke. In our study, two‐photon microscopy imaging was used in vivo to evaluate leukocyte‐endothelial cell interactions such as adhesion and aggregation in stroke mice. It was in vivo clearly observed that these leukocytes obstructed cerebral microvessels and then impaired blood reperfusion (no‐reflow). In other words, leukocyte‐endothelial cell interactions can lead to no‐reflow after recanalization in stroke. Notably, upon treatment of TXL, leukocyte‐endothelial cell interactions were significantly inhibited, and then the obstruction of microvessels was alleviated. These data suggest that TXL improves on‐reflow after recanalization in stroke in association with suppression of leukocyte‐endothelial cell interactions.

Leukocytes play a prominent role in inflammation and immune responses. Clinical data revealed that increased leukocytes at 24 h after intravenous thrombolysis could predict unfavorable prognosis in patients with acute brain infarction.^21^ In experimental cerebral ischemia, leukocytes such as neutrophils, monocytes and macrophages were increasingly recruited to not only the ischemic hemisphere but also to the contralateral hemisphere at 24 h after stroke.^22^ To identify subtypes of adherent and aggregated leukocytes in ischemic stroke, different immune cells in peripheral blood were examined by flow cytometry at 24 h and 72 h after tMCAO. Our results showed that ischemic stroke led to leukocyte subtype changes in peripheral blood mainly including neutrophils, lymphocytes, N/L, NK cells, Tregs, Ts cells, Th1 and Th2 cells, Th1/Th17, Th2/Th17, B cells, macrophages, and DCs. Interestingly, most of their changes were exerted in a time‐dependent manner such as Tregs, Ts cells, NK cells, Th1/Th17, Th2/Th17, B cells, macrophages, and DCs. Of note, TXL could downregulate percentages of neutrophils, Tregs, Ts cells, Th1 cells and Th2 cells, N/L, Th1/Th17, and Th2/Th17, and upregulate NK cell percentage after tMCAO.

As the first leukocytes to arrive in ischemic lesion, neutrophils play a critical role in postischemic stroke inflammation. Neutrophils could extend inflammatory response via releasing cytokines and chemotactic factors, damage endothelial cells and obstruct microcirculation.^23^ Clinical study has revealed that leukocyte and neutrophil counts are increased on the first day after acute ischemic stroke.^24^ Accordingly, reducing neutrophil in peripheral blood could improve stroke outcomes.^25^ Cerebral ischemia leads to augmentation of brain infiltration and activation of neutrophils and lymphocytes in type 2 diabetic mice.^26^ Consistent with studies above, our work has demonstrated that neutrophil ratio is remarkedly increased at acute stages after tMCAO, and TXL can reduce neutrophil ratio in ischemic stroke. Reduced lymphocyte counts in patients with leukocytosis could be also regarded as a predictor of unfavorable outcomes in ischemic stroke.^27^ More importantly, N/L could predict cerebral edema and clinical outcomes, and thus be considered as a prognostic biomarker of early stroke with reperfusion therapy.^28^, ^29^ A retrospective study found that high N/L is considered as a predictor of poor stroke prognosis, while low one is a predictor of favorable outcomes at the early stage of stroke.^30^ Well in line with studies above, our research revealed that cerebral ischemia with reperfusion lead to reduced lymphocyte ratio and increased N/L. TXL could reduce N/L but does not influence lymphocyte ratio after stroke.

Tregs play a protective role in ischemic stroke.^31^, ^32^ Experimental study demonstrated that increased Tregs could promote white matter repair and neurological recovery during the chronic phase of ischemic stroke.^33^ In contrast to these reports, our data showed that the ratio of Tregs was firstly decreased, and then increased after tMCAO in a time‐dependent manner. Interestingly, TXL reduced the parameter at 72 h and did not affect it at 24 h. The results suggest excessive Tregs may have a detrimental effect on stroke recovery, and TXL could rectify the imbalance of Tregs. It is also explained that TXL could promote the migration of Tregs from peripheral blood to ischemic brain to exert protective actions. Ts cells have an effect of immunosuppression. Experimental and clinical data have revealed that Ts cells play a protective role in ischemic stroke.^34^, ^35^ In contrast to these studies, we found an increased ratio of Ts cells at 72 h after tMCAO. The ratio of Ts cells was constant at 24 h. TXL could reduce the ratio of Ts cells at 72 h. These data suggest that excessive Ts cells suppressing immune responses may be unfavorable for stroke recovery, and TXL could correct the excessively enhanced immunosuppression from Ts cells. It is also interpreted that TXL could promote the migration of Ts cells from peripheral blood to ischemic brain to exert protective actions. Th1 and Th17 cells could promote inflammation, while Th2 cells inhibit inflammatory responses. In experimental brain ischemia, the frequency of Th1 and Th17 cells, and Th1/Th2 ratio were significantly increased, and Th2 ratio was constant after stroke.^36^ Clinical studies have demonstrated that elevated Th17 cell ratio and the imbalance between Th1 and Th2 cells in the peripheral blood of ischemic stroke patients are correlated with poor prognosis.^37^, ^38^ Our data are not fully consistent with the studies above. Cerebral ischemia with reperfusion leads to the increase of Th1 and Th2 cell ratio, Th1/Th17, and Th2/Th17, but does not affect Th17 cells at an early phase of stroke. TXL can modulate the balance of these Th cells.

The role of B cells in stroke remains controversial. In experimental stroke, targeting B cells do not influence lesion volume and functional outcomes during the acute phase.^39^ Some reported a deleterious role of B cells on cognition after stroke,^40^ while others found a beneficial effect of the cells on stroke volume and functional outcomes.^41^ In our work, B cell ratio is reduced at early acute phase of stroke. TXL do not influence it. Macrophages play dual roles of both inflammation and anti‐inflammation in ischemic stroke, depending on their cellular phenotypes and stages poststroke. These cells were reported to exert a deleterious effect through producing inflammatory cytokines in cerebral ischemia.^42^ On the other hand, macrophages could clear dead cells and reduce inflammation, and thus promote brain repair after cerebral ischemia.^43^ Consistently with the studies above, our data showed that cerebral ischemia with reperfusion induces an increased M1 and M2 macrophages, and do not influence total macrophages. TXL could upregulate M2 macrophage ratio after stroke.

NK cells could mediate inflammatory responses by cellular cytotoxicity or cytokine release. In experimental brain ischemia, these cells aggravate tissue damage during the acute phase of stroke.^44^ However, clinical data have revealed that NK cell counts are constant or decreased during the acute phase of stroke from different studies.^45^ Consistently with the clinical data above, our work showed that NK cell ratio was decreased at 24 h after tMCAO, while it was constant at 72 h. TXL could antagonize the decreasing process of NK cells to regulate immune reactions at an early stage of stroke due to the fact that the cells could not only promote inflammation, but also modulate immune responses by cross‐talk with other types of immune cells.^45^ Besides, brain ischemia induces DC migration and maturation. Previous study demonstrated that suppression of DC plays a protection role in experimental ischemic stroke.^46^ In our work, the ratios of both plasmacytoid and myeloid DCs are reduced at early acute phase of stroke. TXL do not influence them.

Previous research has demonstrated that leukocyte‐endothelial cell interactions are mainly mediated by the elevated adhesion molecules and chemokines.^9^ To do so, activated endothelial cells could express numerous adhesion molecules and chemokines mediating interactions with leukocytes, such as P‐selectin, E‐selectin, ICAM‐1, and CCL‐2.^47^ Among them, chemokines CCL‐2, CCL‐3, and CCL‐5 participate in leukocyte adhesion.^48^, ^49^ CCL‐4 induces lymphocytes transmigration to neurovascular barrier disruption.^50^ CXCL1, as a neutrophil‐specific chemokine, improves neurological outcomes in experimental ischemic stroke.^25^ To explore molecular mechanisms of leukocyte‐endothelial cell interactions and the inhibitory effects of TXL on the interactions after recanalization in stroke, adhesion molecules, and chemokines in plasma and brain were measured by CBA at 24 h and 72 h after tMCAO. Our results have revealed that ischemic stroke results in upregulated expression of adhesion molecules (P‐selectin, E‐selectin, and ICAM‐1) and chemokines (CCL‐2, CCL‐3, CCL‐4, CCL‐5, and CXCL‐1). Impressively, TXL could inhibit the expression of stroke‐induced upregulated adhesion molecules (P‐selectin and ICAM‐1) and chemokines (CCL‐3, CCL‐4, CCL‐5, and CXCL1).

## 5 CONCLUSIONS

In conclusion, our study has demonstrated that leukocyte‐endothelial cell interactions obstructing brain microvessels is an important cause of no‐reflow after arterial recanalization in ischemic stroke. These leukocytes may mainly include neutrophils, lymphocytes, Tregs, Ts cells, Th1 and Th2 cells, B cells, macrophages, NK cells and DCs. Multiple adhesion molecules and chemokines mediate leukocyte‐endothelial cell interactions. Of interest to therapeutic evolution, TXL can alleviate no‐reflow, protect neurons and thus improve neurological deficits in ischemic stroke with arterial recanalization. The potential mechanism of TXL alleviating no‐reflow is associated with suppressing leukocyte‐endothelial cell interactions by modulating multiple leukocyte subtypes and inhibiting the expression of multiple adhesion molecules and chemokines. Our findings provide a novel therapeutic strategy for no‐reflow after arterial recanalization in ischemic stroke and comprehensive experimental evidence to support application of TXL in the treatment of no‐reflow in the future.

## AUTHOR CONTRIBUTIONS

Ying Gao and Shen Liu designed this study. Yannan He and Qiushuo Jin provided the technical support. Shen Liu, Lingbo Kong, and Dahe Qi performed the experiment. Zhao xu Zhang and Xiangjia Qi took part in data collection and analysis. Shen Liu wrote the manuscript, and Ying Gao revised it. All authors read and approved the final manuscript.

## CONFLICT OF INTEREST STATEMENT

All authors declare no conflict of interest.

## Data Availability

The datasets generated during the current study are available from the corresponding and first author on reasonable request.
